# Only a boost away from re-entry

**DOI:** 10.1007/s12471-017-0990-3

**Published:** 2017-04-24

**Authors:** E. Ströker, C. de Asmundis, G. B. Chierchia, P. Brugada

**Affiliations:** Heart Rhythm Management Centre, UZ Brussel-VUB, Brussels, Belgium

A 31-year-old patient with Brugada syndrome presented at our clinic after an electrical shock from his dual-chamber implantable cardioverter-defibrillator (ICD – Biotronik Iperia), implanted in secondary prevention of sudden cardiac death (ventricular fibrillation zone > 214 bpm, detection counter 18/24; monitor ventricular tachycardia zone > 176 bpm with supraventricular tachycardia discriminators). A regular tachycardia was stored with a total absence of atrial electrograms (defective atrial lead), but a supraventricular origin was suspected (similar QRS morphology on ventricular electrograms compared with sinus rhythm). As the electrophysiological study could not induce any arrhythmia nor evoke dual atrioventricular nodal physiology, the atrial lead was replaced in consideration of potential future events. Two months later, he received another ICD shock, again after exercise-induced palpitations with neck fullness.

Fig. 1 shows the new episode as stored in the device memory. What would be your diagnosis and your next step or treatment?

**Fig. 1:**
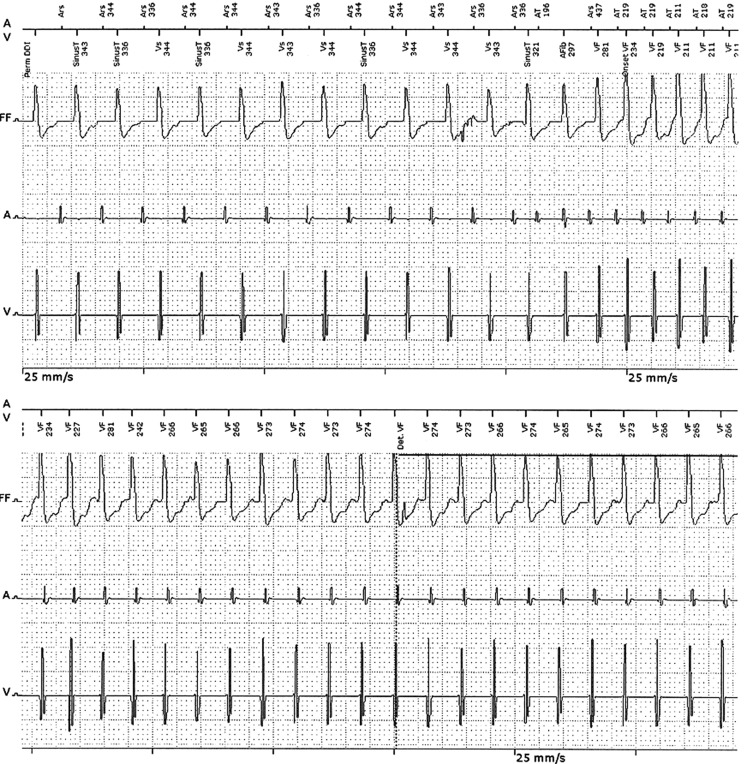
Stored electrograms (far-field, atrial, ventricular) reflecting the arrhythmia episode (continued on lower panel), leading to an inappropriate implantable cardioverter-defibrillator shock (not shown)

## Answer

You will find the answer elsewhere in this issue.

